# Molecular basis for chirality-regulated Aβ self-assembly and receptor recognition revealed by ion mobility-mass spectrometry

**DOI:** 10.1038/s41467-019-12346-8

**Published:** 2019-11-06

**Authors:** Gongyu Li, Kellen DeLaney, Lingjun Li

**Affiliations:** 1 School of Pharmacy, University of Wisconsin-Madison, Madison, WI 53705 USA; 2 Department of Chemistry, University of Wisconsin-Madison, Madison, WI 53706 USA

**Keywords:** Peptides, Protein aggregation, Mass spectrometry, Self-assembly

## Abstract

Despite extensive efforts on probing the mechanism of Alzheimer’s disease (AD) and enormous investments into AD drug development, the lack of effective disease-modifying therapeutics and the complexity of the AD pathogenesis process suggest a great need for further insights into alternative AD drug targets. Herein, we focus on the chiral effects of truncated amyloid beta (Aβ) and offer further structural and molecular evidence for epitope region-specific, chirality-regulated Aβ fragment self-assembly and its potential impact on receptor-recognition. A multidimensional ion mobility-mass spectrometry (IM-MS) analytical platform and in-solution kinetics analysis reveal the comprehensive structural and molecular basis for differential Aβ fragment chiral chemistry, including the differential and cooperative roles of chiral Aβ N-terminal and C-terminal fragments in receptor recognition. Our method is applicable to many other systems and the results may shed light on the potential development of novel AD therapeutic strategies based on targeting the D-isomerized Aβ, rather than natural L-Aβ.

## Abstract (web summary)

Chiral inversion of amino acids is thought to modulate the structure and function of amyloid beta (Aβ) but these processes are poorly understood. Here, the authors develop an ion mobility-mass spectrometry based approach to study chirality-regulated structural features of Aβ fragments and their influence on receptor recognition.

## Introduction

As a spontaneous post-translational modification in living systems, naturally occurring D-amino acid substitution has been observed in many neurodegenerative disease-related peptides and proteins^1^. Specifically, extensive efforts have been devoted to unveiling the amino acid chiral inversion of amyloid beta (Aβ), which is believed to be involved in the pathological development, e.g., long-term potentiation disruption, of Alzheimer’s disease (AD)^2–5^. With continuous development of improved analytical techniques, the chiral inversion of Aβ has been experimentally demonstrated to be programmed by an age-dependent pathway^2,5^. Aspartic acid (D) and serine (S) are the two most common isomerization residues found in AD tissue, that is, D-aspartic acid (dD Aβ) and D-serine (dS Aβ)^5–7^. Notably, only a small portion (e.g., less than 10%) of Aβ species was found to be D-isomerized^6^. This fact along with the subtle structural changes induced by amino acid chiral inversion would lead to overlooking this important modification and its biological consequences^8,9^, such as the role of Aβ chiral chemistry in regulating amyloid oligomerization, fibrillation and plaque deposition which are closely linked to the pathological development of AD. In addition, most D-isomerized, biologically-active peptides/proteins are more resistant to enzymatic degradation in living systems^10–12^, which makes clearance more difficult and might result in a higher self-assembly/oligomerization propensity. In other biological systems, the presence of D-amino acid containing peptides (DAACPs) as signaling molecules have also been well documented^1,8,13,14^. For example, Sweedler and coworkers reported on peptides with a single amino acid chiral inversion acting as signaling molecules, which exhibit enhanced biological functions in many cases, e.g. dramatically higher receptor-binding affinity and selectivity^8^. It is worth noting that Aβ with chiral inversion at all amino acids was reported to have less toxicity^3^, while peptides (e.g., neuropeptides and Aβ fragments) with single/partial amino acid chiral inversion tend to be essential for many biological activities or possess higher toxicity^8,9^. Furthermore, chirality has been shown to have a profound effect on aggregation kinetics and cytotoxicity^3,15^. To elucidate the poorly-understood mechanisms underlying chiral chemistry and clarify its controversial role in disease progression and therapeutic treatment, interrogation of the effect of chiral residue(s) on peptide self-assembly/oligomerization process is highly desirable. Furthermore, the impact of chirality on receptor recognition and binding process of these biologically active peptides, including Aβ, should be investigated. Understanding these molecular changes and underlying mechanisms of chiral chemistry is very important but challenging for single/partial amino acid chiral inversion due to the analytical difficulty in distinguishing subtle structural differences induced by single/partial D-amino acid substitution^12^.

Ion mobility-mass spectrometry (IM-MS) has been growing as a powerful tool for peptide and protein complex structural analysis^16–19^. IM-MS has unique advantages in analytical speed, sensitivity and sample consumption over many other biophysical techniques and can simultaneously provide molecular and structural information of proteins and peptides. Since early IM-MS for chiral peptide analysis was developed in 2000 with relatively low separation efficiency^20^, numerous instrumental modifications on IM-MS have significantly improved the resolving power such as the structures for lossless ion manipulations (SLIM)^21,22^. Recent commercial IM-MS setups are largely based on drift-tube ion mobility spectrometry (DTIMS), traveling-wave ion mobility spectrometry (TWIMS) and trapped ion mobility spectrometry (TIMS) instruments with high mass range and abundant structural information^14,23–25^. Previously, we have developed a versatile method to localize the D-amino acid residues in a wide range of DAACPs by monitoring the structural changes of fragment ions generated from collision-induced dissociation^14^. Notably, the resolving power of D/L epimers on commercial IM-MS instruments is still a key limiting factor. Although substantial progress has been made over the past several years^20,26–28^, the development of rapid and effective analytical strategies for structural discrimination of optically impure peptide epimers is still in great demand.

In this study, through the development of an IM-MS-based integrative chirality anatomy platform (iCAP), we aim to reveal the epitope region-specific, chirality-regulated Aβ fragment structural features. The co-D-isomerization of Asp- and Ser-residues not only induces larger structural changes of both monomers and oligomers, but also exerts greater structural effects on long N-termini in recognizing receptors (e.g., serum albumin) while single Asp- or Ser-residue D-isomerization affects mostly the binding behaviors of C-termini to receptor (e.g., transthyretin). Our results may provide supplementary molecular insights into the recently reported Aβ epitope region-specific response to external stimulus^29^, as well as the potential development of novel AD therapeutic strategies based on targeting the co-existing D-isomerized Aβ, rather than targeting solely the natural L-Aβ.

## Results

### The conception and workflow for iCAP

**Fig. 1:**
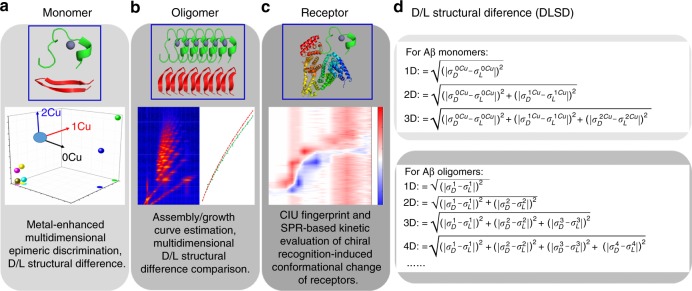
The overall conception of iCAP. **a** Metal-enhanced multidimensional epimeric discrimination is used to amplify the D/L structural differences (DLSDs) of Aβ monomers. **b** Discrimination of chiral Aβ fragment oligomers by assembly/growth curve based multidimensional DLSD comparisons. **c** Elucidation of chiral Aβ-receptor recognition by CIU fingerprint and SPR-based kinetic evaluation. **d** The calculation equations for DLSD. $\sigma _D^{0\mathrm{Cu}}$/$\sigma _L^{0\mathrm{Cu}}$: CCS values of apo-Aβ fragment; $\sigma _D^{1\mathrm{Cu}}$/$\sigma _L^{1\mathrm{Cu}}$: CCS values of [Aβ + Cu(II)] complex; $\sigma _D^{2\mathrm{Cu}}$/$\sigma _L^{2\mathrm{Cu}}$: CCS values of [Aβ + 2Cu(II)] complex. $\sigma _D^1$/$\sigma _L^1$/$\sigma _D^2$/$\sigma _L^2$/…$\sigma _D^n$/$\sigma _L^n$: CCS values of Aβ oligomers (aggregation number = *n*). All charge states were considered during DLSD calculation

**Fig. 2:**
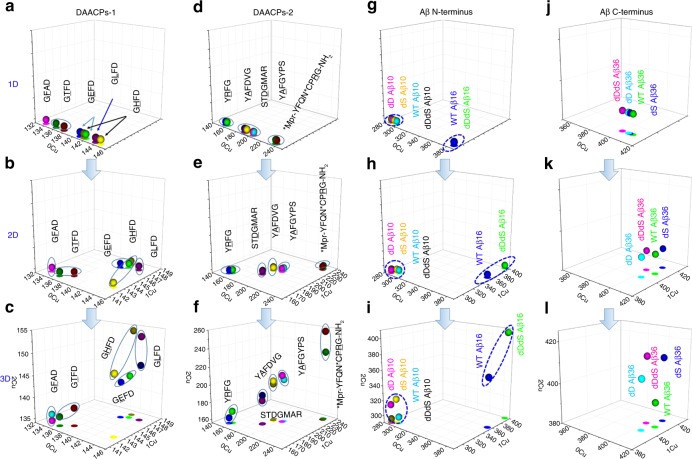
Metal-enhanced multidimensional epimeric monomer discrimination. Group 1 (**a**–**c**): short neuropeptides **1**–**5**; Group 2 (**d**–**f**): long neuropeptides **6**–**10**; Group 3 (**g**–**i**): Aβ N-terminal fragments **11**–**12** and Group 4 (**j**–**l**): Aβ C-terminal fragments **13**. With the increase of separation dimensions (sequential binding with Cu^2+^), significantly improved separation efficiency is achieved for a wide range of neuropeptides and Aβ fragments (30 in total). All CCS data denote average values (*n* = 3). Source data are provided as a Source Data file

**Fig. 3:**
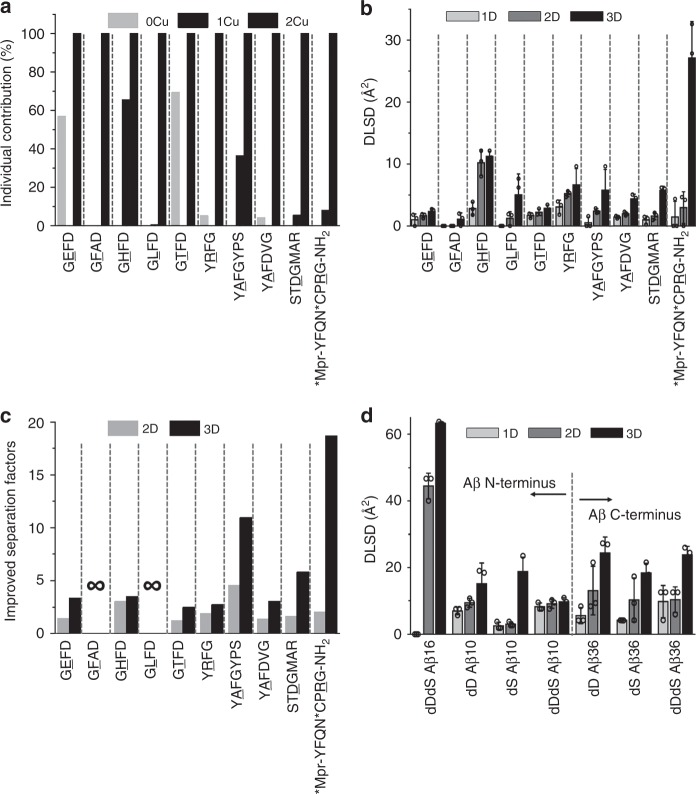
Characterization of the separation efficiency of iCAP. **a** Individual contribution ratio is calculated by normalizing the CCS differences of D/L-peptides (0Cu- and 1Cu-bound complexes) to 2Cu-bound complexes. In all cases, the individual contributions of 2Cu-bound complexes are thus defined as 100%. Results indicate the necessity of employment of multidimensional separation. **b** DLSD values for a wide range of chiral neuropeptides under multidimensional conditions. **c** Improved separation factors for each DAACP group calculated through dividing the 2D-DLSD and 3D-DLSD values by 1D-DLSD. ∞, unresolved peptides (GFAD/GLFD) are effectively separated with DLSD calculation even though they are not quantifiable by using improved separation factor due to 1D-DLSDs for GFAD and GLFD being 0. **d** DLSD values for chiral Aβ fragments. Peptide groups are separated by green dashed lines. All error bars denote S.D.; *n* = 3 biologically independent samples. Box charts are shown with mean values. Source data are provided as a Source Data file

Facing the analytical challenges and limitations, herein, we aim to develop an integrated analytical platform, iCAP, based on multidimensional IM-MS measurements to simultaneously provide molecular and structural insights into the chiral chemistry of Aβ fragment self-assembly/oligomerization and its receptor recognition. In addition to the versatile compatibility with any other IM-MS instruments and broad applicability to the study of other protein aggregation systems, iCAP can simultaneously address three challenges: (1) Rapidly and efficiently distinguish D/L-amino acid containing Aβ fragment monomers through metal-enhanced multidimensional epimeric discrimination (Fig. 1a); (2) Directly read out the Aβ fragment oligomerization processes using growth curves and evaluate the chiral effect involved in the oligomerization process (Fig. 1b); (3) Reliably visualize the chiral recognition-induced structural changes of receptors based on collision-induced unfolding (CIU)-IM-MS measurements and surface plasmon resonance (SPR)-based kinetic analysis (Fig. 1c). CIU-IM-MS has been demonstrated to be useful to identify subtle structural changes of a wide range of gas-phase protein ions^30,31^. The solution-phase kinetic assessments further validated the rapid gas-phase CIU-IM-MS results. The advantages of iCAP originate from both the rationally-designed, multidimensional chiral amplification strategy through metal-binding events (Fig. 1a) and a newly-proposed parameter, D/L structural difference (DLSD, right panel in Fig. 1), to quantitatively characterize the effect of amino acid chirality on Aβ fragment monomer structure and oligomerization pathway. In addition, the root mean square deviation, RMSD, as calculated from CIU difference plots, and CIU50, as calculated from CIU fingerprints, also contribute significantly to the quantitative characterization of the chiral inversion-induced structural recognition of Aβ receptors.

### Discrimination of chiral Aβ fragment monomers by iCAP

As a first step in iCAP, we need to discriminate D/L epimeric monomers. D/L peptide epimer separation is also vital for peptide functional interrogation and peptide-based drug discovery targeting several neurodegenerative diseases. Typically, epimers can be separated with a chiral column by HPLC or capillary electrophoresis^8,32^. In this study, we provide a general, effective multi-dimensional strategy as a faster alternative to HPLC separation. A wide range of chiral centers (Table 1) have been evaluated by the three-step iCAP. As a proof-of-concept experiment, we selected partial D-amino acid substitution of Asp and Ser, two of the most commonly-observed naturally-occurring chiral inversion sites in Aβ, as our chirality core. To study the domain-specific chiral effects, three types of Aβ peptides comprising N-terminal and C-terminal fragments are tested, Aβ (1–10), Aβ (1–16) and Aβ (17–36) (Table 1). The analysis of these Aβ peptide fragments facilitates understanding the domain/epitope region-specific structural features regulated by amino acid chirality. Interestingly, the epitope region-specific structural features and the associated differential response to external stimulus for different epitope regions via rational design of antibodies targeting to different domains/epitopes of Aβ peptide have been recently highlighted^29,33^. It appears that, from this recent study, the N-terminal region of Aβ is responsible for causing an inflammatory response in microglia cells through formation of larger soluble aggregates, while the C-terminal region of Aβ tends to induce membrane permeability through formation of small aggregates^29^. Therefore, it would be of great interest to study the epitope region-specific, chirality-regulated structural features of Aβ peptides.

**Table 1:** Information for 30 chiral peptides (13 groups) including neuropeptides with single D-amino acid (DAACPs) and Aβ with D-isomerized Asp and Ser-residues (dD/dS Aβ)

Group	Peptide	Sequence	Chiral center	Notes
1	GEFD	GEFD	E (Glu)	DAACP
2	Achatin I	GFAD	F (Phe)	DAACP
3	GHFD	GHFD	H (His)	DAACP
4	GLFD	GLFD	L (Leu)	DAACP
5	GTFD	GTFD	T (Thr)	DAACP
6	Dermorphin 1–4	YRFG	R (Arg)	DAACP
7	Ala-Dermorphin	YAFGYPS	A (Ala)	DAACP
8	Deltorphin I	YAFDVVG	A (Ala)	DAACP
9	STDGMAR	STDGMAR	D (Asp)	DAACP
10	Deamino vasopressin	^a^Mpr-YFQN*CPRG-NH_2_	R (Arg)	DAACP
11	Aβ (1–10)	DAEFRHDSGY	D (Asp), S (Ser)	dD/dS/dDdS Aβ
12	Aβ (1–16)	DAEFRHDSGYEVHHQK	D (Asp), S (Ser)	dDdS Aβ
13	Aβ (17–36)	LVFFAEDVGSNKGAIIGLMV	D (Asp), S (Ser)	dD/dS/dDdS Aβ

^a^Disulfide bond between Mpr and Cys. Mpr, 3-mercapropionic acid

It has been well-established that metal binding is an important regulating factor during amyloid aggregation^34,35^. In our strategy, the structural differences between D/L peptide epimers are intentionally amplified by metal coordination, as implicated by recent systematic IM-MS studies on metal-peptide interaction^36^. Furthermore, a data visualization method is developed through three-dimensional (3D) scattering of collisional cross-section (CCS) values of metal-bound complexes in a 3D space, which is inspired by a previous study on multidimensional analysis of glucose isomers^37^. Herein, we rationally select the following binding ions as the indicators in our strategy for the multidimensional separation, [P + H]^+^, [P + M-H]^+^, [P + 2M-3H]^+^. Due to the differences in coordination ability with metals, D/L peptide epimers show diverse binding ratios and, more importantly, the resultant peptide-metal complexes bear even more striking structural differences. All these binding events are monitored using a commercial TWIMS-MS instrument.

To demonstrate the effectiveness of iCAP in discriminating chiral monomers, we firstly introduce ten pairs of neuropeptide D/L epimers with single amino acid chiral inversion (Table 1). As shown in Table 1, we apply iCAP to 9 amino acid chiral centers in total. After data collection and CCS calculation, we visualize each peptide in a 3D space with scatter plots and XY projections. Each peptide is characterized by a spatial vector, where each coordinate (x, y, z) corresponds to the CCS value of [P + H]^+^, [P + Cu(II)-H]^+^, and [P + 2Cu(II)-3H]^+^, respectively. As a result, several 3D plots comprising CCS values for the D/L peptide epimers in Table 1 have been obtained and shown in Fig. 2. We select Cu^2+^ as a first choice of metal because it has a unique isotopic distribution, moderate binding affinity to most peptides and is widely involved in various biological systems including neurological diseases. The theoretically calculated and experimentally measured isotopic distributions of peptide-Cu(II) binding complexes match each other perfectly (Supplementary Fig. 1).

In 1D analysis, only normal CCS measurements of protonated peptides are used, and no metal binding is introduced, and as expected, the spatial distribution is congested (Fig. 2a, d, g and j). For 2D experiments, a second component is included, the CCS of 1Cu-bound peptide complex. Interestingly, the spatial distribution has been expanded (Fig. 2b, e, h and k), such as for GLFD. The spatial distribution can be even further expanded with the addition of a third component (Fig. 2c, f, i and l), CCS values of 2Cu-bound peptide complexes. Therefore, with the increase of copper binding along with the increase of separation dimension, the separation efficiency between wild type and chiral inversed peptides is increased remarkably. Therefore, the 3D scattering method has provided a visualization method that enables the direct enhancement of the separation even without any instrumental modifications.

To quantitatively characterize the separation efficiency, we directly measure the spatial distance in 3D space in Fig. 2 as implicated by the basic concept in solid geometry. The spatial distance in the 3D scattering plot is specifically used to characterize DLSD and is then plotted as a bar graph in Fig. 3. Based on the individual contribution results in Fig. 3a, in all cases the third dimension contributes the most, but in some cases the first and second dimension also contribute in some way. Thus, multi-dimensional separation in our strategy is crucial because the combined 3D separation helps to maximize the performance of iCAP. Figure 3c shows the improved separation efficiency for various D/L peptide epimers. It clearly shows that the structural differences between D/L epimers are significantly amplified for most peptides (Fig. 3b/d). For example, peptide deamino vasopressin gives a DLSD value of 0.7 Å^2^ in 1D separation while the DLSD value in 3D separation is as high as 23.7 Å^2^. In other words, the separation efficiency of peptide deamino vasopressin has been improved by ~19-fold. Interestingly, some cases, peptides like GFAD/GLFD/Aβ (1–16), that could not be distinguished under normal IM-MS measurements, are effectively differentiated by the iCAP strategy as indicated by the significantly elevated DLSD values. Figure 3d shows the DLSD measurements from 10 chiral Aβ N-terminal and C-terminal fragments, and it seems that the D-isomerization induces much greater structural change of N-terminal fragment than that of C-terminal fragment. These data demonstrate the versatility of using DLSD to quantitatively characterize the separation efficiency of these peptide epimers, and the differential role of chirality of Aβ N-terminal and C-terminal can be inferred.

### Discrimination of chiral Aβ fragment oligomers by iCAP

**Fig. 4:**
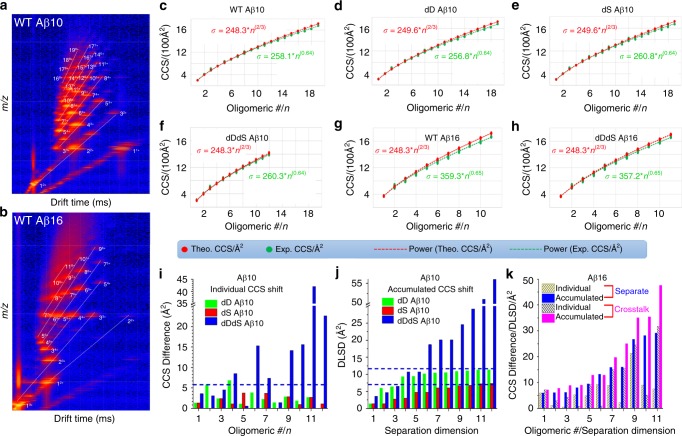
iCAP for distinguishing chiral Aβ fragment oligomers. **a**, **b** Representative Driftscope maps for WT Aβ (1–10) and Aβ (1–16). **c**–**h** Growth curves for various Aβ fragments. For comparison, both isotropic growth-based prediction and IM-MS measurement-based experimental results have been shown. **i** CCS differences as a function of oligomeric number of Aβ (1–10) as calculated simply by deduction of CCS values from individual WT Aβ oligomers. **j** DLSD values as a function of separation dimension for accumulated Aβ (1–10) oligomers as calculated by accumulating the CCS differences in (**i**) using functions in Fig. 1. **k** Individual CCS differences (yellow and gray bars that are made of oblique lines) as a function of oligomeric number of Aβ (1–16) and accumulated DLSD values (blue and magenta bars) as a function of separation dimension for separate (WT and dDdS peptides were separately analyzed) and crosstalked (mixing WT and dDdS peptides) Aβ (1–16) oligomers. Aβ (1–10) and Aβ (1–16), 600 µM. Buffer, 10 mM NH_4_OAc. Crosstalking experiments, final concentrations of individual WT Aβ (1–16) and dDdS Aβ (1–16), 300 µM. Source data are provided as a Source Data file

One of the AD hallmarks is extracellular amyloid plaque deposition in the brain, which is believed to be linked to the Aβ self-assembly/oligomerization^38^. For step 2 in iCAP, we use the same chiral amplification concept as in step 1, measurements of structural differences between wild type and chiral inversed peptides as a function of oligomeric number and separation dimension, to directly read out the chiral effects in Aβ self-assembly/oligomerization. Figure 4 showcases the use of iCAP for distinguishing Aβ fragment oligomers. The left panels (Fig. 4a, b) depict the typical Driftscope data of Aβ fragment oligomerization. Distinct Aβ fragment oligomerization can be traced as indicated by the oligomers with various charge states, for example, dimer with two and three charges and trimer with three and four charges. We then generate the growth curves (Fig. 4c–h) of these oligomers by plotting the CCS values against the oligomeric number. In Fig. 4c–h, the theoretical CCS values represent the theoretical structures of oligomers following isotropic growth pathway; the experimental CCS values represent measured oligomer structures by using IM-MS. To track the potential chiral effects on oligomer growth pathway, both the theoretical and experimental CCS values of each oligomer are fitted into a power relationship as a function of oligomeric numbers. Aβ fragments tested here follow an isotropic assembly pathway as indicated by the similarities between the predicted isotropic growth curve and IM-MS-measured growth curve. To examine the reliability of the isotropic growth curve, we then test a known isotropic growth model of YGGFL^39^. The formation of granular and isotropic aggregate can be indicated by the corresponding mass spectrum (Supplementary Fig. 2), concentration-dependent aggregation (Supplementary Fig. 3) and isotropic growth curve (Supplementary Fig. 4). Under isotropic growth conditions, the CCS value (*σ*) should follow the growth function: *σ* = *σ*_1_*n*^*2/3*^, (*σ*_1_, the CCS value of the peptide monomer)^39^. Interestingly, both YGGFL (Supplementary Fig. 4) and Aβ N-terminal fragments (Fig. 4c–f) follow a function that is very close to isotropic growth. The chiral inversion at Asp and Ser residues does not significantly change the self-assembly pathway of Aβ (1–16) and Aβ (1–10), as indicated by the isotropic growth curve (Fig. 4d–f and h) fitting from IM-MS data.

However, the IM-MS data of growth curve (Fig. 4c–h), individual CCS differences (Fig. 4i) and DLSD value calculations (Fig. 4j) indicate that Asp-isomerization induces greater structural changes of Aβ oligomers compared to Ser-isomerization, both of which show less structural changes than co-isomerization of Asp and Ser residues. For Aβ (1–10), as shown in the Driftscope data (Supplementary Fig. 5) and growth curve (Fig. 4c–f), co-isomerization of the chiral centers of Asp and Ser results in the formation of smaller oligomer sizes, only 12mer compared with 19mer for wild type. The chiral effects seem to be compensated with the increase of the peptide length, as indicated by Aβ (1–16) (Fig. 4g–h). Figure 4i, j is the individual CCS differences of Aβ (1–10) oligomers and corresponding accumulated DLSD results as a function of separation dimension, respectively, as calculated from the isotropic growth curves for these chiral oligomers using functions in the right panel of Fig. 1. As shown in Fig. 4i, while both Asp- and Ser-isomerization of Aβ (1–10) have resulted in DLSD of no more than 5 Å^2^, co-isomerization of Asp and Ser seems to induce more structural change for multiple oligomers especially for those with higher oligomeric numbers. Interestingly, benefiting from the concept of multidimensional separation of iCAP, data of accumulated DLSD values of Aβ (1–10) oligomers in Fig. 4j give rise to a distinct trend for the increasing chiral effect in the order of single Ser-isomerization (DLSD of no more than 7 Å^2^), single Asp-isomerization (DLSD of no more than 12 Å^2^) and co-isomerization of Asp- and Ser-residues (most DLSDs of more than 25 Å^2^), respectively.

In addition, we also test the crosstalking chiral effects of Aβ fragments isomerized at Asp and Ser residues as the wild type Aβ and D-isomerized Aβ often coexist in nature rather than being individually present in cellular environments. Thus, it is more interesting to interrogate the chiral effects with the presence of several forms of Aβ peptides. Crosstalking chiral effects are investigated through mixing different forms of Aβ peptide fragments with various chiral Asp- and Ser-residues. Supplementary Fig. 6 shows the representative Driftscope maps, oligomerization curves and corresponding DLSD changing trends for various crosstalked Aβ (1–10) oligomers. The enhanced crosstalking chiral effects on oligomerization can be revealed by mixing WT Aβ (1–10) with dD Aβ (1–10) and/or dS Aβ (1–10), where the ultimate DLSDs are increased from 12 and 7 Å^2^ (Fig. 4j) to 20 and 14 Å^2^ (Supplementary Fig. 6) for dD Aβ (1–10) and dS Aβ (1–10), respectively. We then study the co-isomerization of Asp- and Ser-residues on a longer fragment, Aβ (1–16). Data derived from Aβ (1–16) in Fig. 4k showcase the dependency of individual CCS differences on oligomeric numbers (yellow and gray bars) and the dependency of accumulated DLSD values on separation dimension (blue and magenta bars). Direct comparisons between individual CCS differences as a function of oligomeric number and corresponding accumulated DLSD values as a function of separation dimension for each oligomer suggest the much greater distinctly changing trend of monotonic elevation relationship, which further supports the effectiveness of iCAP for chiral discrimination. More interestingly, the accumulated DLSD values derived from separate dDdS Aβ (1–16) groups (blue bars in Fig. 4k) are generally less than the ones from crosstalked Aβ (1–16) groups (magenta bars in Fig. 4k). This difference between separate and crosstalked (1–16) has been observed with almost all oligomers except 6-mer, and the difference seems to increase with higher oligomeric numbers. Collectively, iCAP results and crosstalking experiments have indicated that after mixing the D-isomerized fragment with wild type Aβ (1–16), the chiral effect exerts an even greater impact on its self-assembly/oligomerization behavior as indicated by the much larger DLSD values for crosstalked groups than separate groups.

### Elucidation of chiral Aβ-receptor recognition by iCAP

Peptides/proteins do not exert their functions individually^40–43^. Instead, many of them exert functions via dynamic interactions with individual or multiple partners existing in the surrounding environment^41,44,45^. Therefore, examining the chiral effects on Aβ-receptor recognition is desired. As a last step of iCAP, we employ in-solution kinetics analysis and gas-phase CIU-IM-MS to interrogate chiral Aβ fragment recognition of their receptors (including HSA/BSA/mTTR/TTR, more information can be found in Supplementary Table 1). As shown in Table 2, our OpenSPR results for full sequence Aβ are consistent with previous ITC reports^46^. For the chiral Aβ C-terminal fragments (Table 2), D-isomerization results in reduction of both binding affinity and binding kinetics between TTR and Aβ (17–36). For example, the binding affinities of single D-isomerized and co-isomerized Aβ (17–36) fragments are ~10 folds and ~8 folds less than that of the WT Aβ (17–36) fragment in recognition of their receptor, tetrameric TTR, respectively. Meanwhile, the binding dissociation rate constants of single D-isomerized and co-isomerized Aβ (17–36) fragments are ~15 folds and ~13 folds higher than the WT Aβ (17–36) fragment in recognition of their receptor, tetrameric TTR, respectively. Surprisingly, dDdS Aβ (17–36) shows similar kinetic behaviors to the full-length Aβ (1–40) in recognition of receptor TTR as indicated by their similar binding affinity (*K*_D_), dissociation constant (*K*_d_) and association constant (*K*_a_) values. These OpenSPR results suggest the effect of chirality in differential kinetic regulation of receptor recognition of Aβ (17–36) fragments, where micromolar range of binding affinity indicates weak binding between Aβ (17–36) fragments and TTR.

**Table 2:** In-solution kinetics analysis of chiral TTR-Aβ interactions via OpenSPR (All data in this study represent average values from three biologically independent samples, ± indicates SD)

Aβ	K_D_ (µM)	K_d_ (1/s) × 10^2^	K_a_ (1/(M*s)) × 10^−2^	Method
Aβ (1–40)	24 ± 12	–	–	ITC (human TTR), ref. ^46^
Aβ (1–40)	16 ± 7	–	–	ITC (mouse TTR), ref. ^46^
Aβ (1–40)	435 ± 10	–	–	Fluorescence, ref. ^60^
Aβ (1–40)	30.8 ± 2.8	7.3 ± 1.0	24.5 ± 4.8	OpenSPR, this study
Aβ (1–42)	73.6 ± 13.3	12.5 ± 0.8	18.2 ± 2.8	OpenSPR, this study
WT Aβ (17–36)	11.5 ± 1.6	0.8 ± 0.1	6.9 ± 0.8	OpenSPR, this study
dD Aβ (17–36)	149.3 ± 21.9	13.2 ± 2.9	9.9 ± 3.2	OpenSPR, this study
dS Aβ (17–36)	118.1 ± 45.2	11.4 ± 2.6	11.6 ± 2.5	OpenSPR, this study
dDdS Aβ (17–36)	83.1 ± 38.0	10.2 ± 1.6	19.3 ± 7.0	OpenSPR, this study

Source data are provided as a Source Data file

**Fig. 5:**
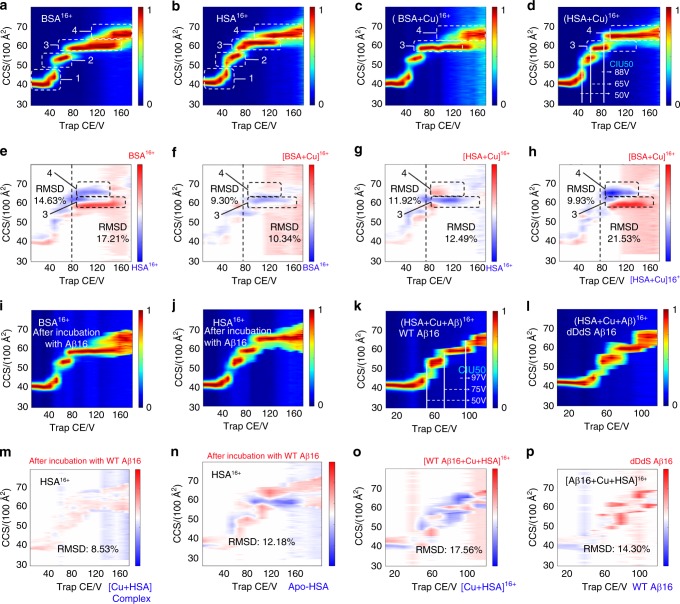
iCAP for distinguishing chiral Aβ (1–16) recognition of receptors. CIU fingerprints: 16 + apo-serum albumin (**a**, **b**), metal-bound serum albumin (**c**, **d**), remaining serum albumin after incubation with Aβ (1–16) and Cu (**i**, **j**), and triple HSA-Aβ-Cu complex (**k**, **l**). CIU difference plots: between BSA and HSA (**e**/**h**), between BSA-Cu complex and BSA (**f**), between HSA-Cu complex and HSA (**g**). The structural changes induced by Aβ (1–16) binding were indicated by CIU50 (**d**/**k**) and CIU difference plots of HSA (**m**/**n**) and HSA-Cu complex (**o**). The differences between WT and dDdS Aβ (1–16) were presented by CIU difference plots of HSA-Aβ-Cu complex (**p**). Four major conformer families (1−4, highlighted in **a**–**d**) are detected. Buffer, 100 mM NH_4_OAc. Concentrations: Aβ (1–16), 15 µM; Cu^2+^, 20 µM; BSA/HSA, 7.5 µM. Source data are provided as a Source Data file

To further reveal the structural details involved in the chiral recognition of receptors, we employ a rapid gas-phase technique, CIU, to follow distinct unfolding pathways when subjected to collisional heating in the gas phase, a useful tool for resolving protein ions with subtle structural variants. As shown in Supplementary Fig. 7, we have compared the binding of wild type and D-isomerized Aβ (1–16) fragments to serum albumin, which is known as a cellular transporter of Aβ in living systems^47,48^. The interactions between Aβ (1–16) fragment and HSA/BSA are confirmed from the gradual release of Aβ fragments from precursor ion activation (Supplementary Fig. 8). Our CIU fingerprint results in Fig. 5a–d, i–l reflect the domain information of serum albumin as indicated by the transitions in the CIU fingerprints through plotting CCS values against collision voltage. Serum albumin is known as a three-domain protein and we observe three transitions from the CIU fingerprints. In well accordance with previous reports^49,50^, four conformer families can be distinctly derived from the CIU features and transitions as shown in Fig. 5a–d. The families are marked with dashed rectangles.

In order to quantitatively characterize subtle structural differences, we apply a function in CIUSuite developed by the Ruotolo group^30,51^, which results in CIU fingerprints and difference plots (Fig. 5e–h, m–p) with two new parameters, CIU50 and RMSD, being used. Higher RMSD values indicate larger differences related to various structural variants. The structural similarity can be further quantified through computing two adjacent RMSD values: one for all CIU data collected at collision voltages less than 80 V (at which the first two conformers can be retained), and another for all CIU data collected above that value (Fig. 5e–h). It is clear from CIU difference plots in Fig. 5e, h that the species differences can be readily distinguished for bovine and human. A differential analysis in Fig. 5e, h shows that CIU features are nearly identical at lower collision energy (below 80 V), as evidenced by the relatively low RMSD values, especially for the Cu-bound complex with RMSD of 9.9. In contrast, the RMSD values above 80 V in CIU difference plots is as high as 21.5, not only strongly supporting the species diversity/biosimilarity between human and bovine, but also suggesting lower gas-phase stability of HSA conformers **3** and **4** than BSA.

By using similar methods, we find that copper binding (Fig. 5f, g) destabilizes serum albumin, as indicated by the CIU difference plots, especially the presence of a much higher ratio of conformer **3** for HSA-Cu complex but with conformer **4** for apo-HSA at the same transition collision voltage. The RMSD values for the zone of conformers **3** and **4** are as high as 12.0%. However, we find that the direct binding of Aβ stabilizes HSA as clearly revealed by the CIU50 comparison in Fig. 5d, k. The CIU50 for conformer **1** to conformer **2** keeps constant at 50 V, but both the CIU50 for conformer **2** to conformer **3** and the CIU50 for conformer **3** to conformer **4** have significantly increased from 65 V/88 V to 75 V/97 V upon binding with Aβ. The stabilization effects on receptors of Aβ are also observed in monomeric transthyretin (mTTR)-recognition events as indicated by corresponding CIU50 values (Supplementary Fig. 9). The CIU50 values might help distinguish subtle structural differences, especially for proteins with several domains, which could be difficult to identify from CIU difference plots (Fig. 5o).

To interrogate the potential competitive interactions between Aβ-Cu(II) and HSA-Cu(II), especially the sequestering effect of HSA on Aβ-Cu(II) binding, we first incubate Aβ N-termini fragments (metal binding domain) and Cu^2+^ in buffer solution at 37 °C for 3 h to ensure the complete binding between each other, which is followed by the addition of apo-HSA into the reaction solution. After another incubation for 3 h, they are then subjected to native CIU-IM-MS analysis where we compare CIU fingerprints between HSA remaining in the reaction solution and separately obtained apo-HSA, and similar comparison with freshly prepared HSA-Cu(II) complex. From CIU difference plots in Fig. 5m, n, we can conclude that after incubation with Aβ fragments, the remaining serum albumin shows more structural similarity to the separately obtained copper-bound complex rather than apo-proteins, as indicated by the much lower RMSD for HSA-Cu(II) complex (8.5, Fig. 5m) than HSA itself (12.2, Fig. 5n). This further suggests serum albumin can sequester copper from Aβ-Cu(II) binding complex, which was also observed in previous direct MS measurements^47^. Figure 5p is the direct comparison of wild type and D-isomerized Aβ (1–16). While D-isomerized Aβ (1–16) fragments tend to be more labile to collision unfolding and bear much broader CCS distribution as indicated by the CCS values of conformer 1 below 40 V in Fig. 5k, l and CIU difference plots in Fig. 5p, no striking difference is observed from the overall structural comparison and unfolding pathway. This is a seemingly reasonable observation due to recent X-ray crystallization results suggesting that the functional receptor-binding region of Aβ might span from residue Val16 to residue Val36 ^52–54^.

Surprisingly, we find that binding of various D-isomerized Aβ (1–10) peptides at different residues (Asp and Ser) induces differential structural changes of the receptor. As shown in Supplementary Fig. 10, the binding complex between HSA and Aβ (1–10) bears same structural domains with HSA itself, as four features are prevalent among CIU fingerprints which is indicative of three domains. To interrogate the chiral effects on receptor recognition of Aβ (1–10), we then make a series of CIU difference plots between the complexes of D-isomerized Aβ (1–10)-HSA and the complex of WT Aβ (1–10)-HSA. As shown in Supplementary Fig. 11, the RMSD values below 40 V are monitored in CIU difference plots for each of these epimeric binding complexes at various charge states from 15+ to 18+. Generally, these RMSD values suggest that the D-isomerization-induced structural changes of the HSA-Aβ (1–10) fragment binding complex follow the orders: dDdS > dD > dS. For example, at charge state of 17+, the RMSD values below 40 V for dDdS, dD and dS are 18.2%, 9.6% and 9.6%, respectively (although the dD and dS is very close to each other in this case). Note that 16+ charge state has exception with dS > dD for RMSD below 40 V although this value for the entire unfolding energy range is still following the trend with dD > dS: 18+, 11.6% (dD) vs 10.5% (dS); 17+, 9.5% (dD) vs 6.0% (dS); 16+, 13.8% (dD) vs 7.2% (dS) and 15+, 13.8% (dD) vs 6.8% (dS). These results (Supplementary Fig. 11) also reveal the stabilities of conformer 1 for the complexes of D-isomerized Aβ (1–10)-HSA are slightly lower than that of WT Aβ (1–10)-HSA as indicated by the slightly increased CCS values at the same collision voltages and by the slightly decreased CIU50 values for D-isomerized Aβ (1–10) species.

## Discussion

Collectively, the iCAP strategy has enabled a more comprehensive evaluation of the complementary molecular evidence for chiral effects of Aβ fragments on monomer structure, oligomerization behavior and potential receptor recognition. By integrating results from each step, we find that co-D-isomerization of Asp- and Ser-residues induces greater structural changes in both monomers (Figs. 2 and 3) and oligomers (Fig. 4) while single D-isomerization at Ser-residue induces the least amount of perturbation on their structures. In addition, it appears that the chiral effects of Asp- and Ser-residues are programmed in an epitope region-specific manner, where co-isomerization of both residues exerts greater structural effects on long N-termini in recognizing receptor (HSA, Fig. 5, Supplementary Figs. 10 and 11) while single Asp- or Ser-residue D-isomerization affects mostly the binding behaviors of C-termini to receptor (TTR, Table 2). This observation corroborates the recently reported epitope region-specific response to external stimulus^29^. Our results may provide supporting molecular evidence for the recent study reporting that small C-terminal aggregates are responsible for inducing membrane permeability while larger N-terminal aggregates are more likely to cause an inflammatory response in microglia cells ^29^.

Recent available structures of Aβ fibrils revealed by NMR studies also suggested the epitope region-specific beta sheet structural features. For example, residues 15–42 of Aβ adopt a double-horseshoe-like cross-β-sheet entity with maximally buried hydrophobic side chains while residues 1–14 of Aβ are partially ordered in a β-strand conformation^55,56^. Here, our findings reinforce the concept that the effect of amino acid chiral inversion should not be underestimated, as we postulate that the chirality of amino acids, especially Asp- and Ser-residues, is very likely to differentially regulate the stabilities of both the C-terminal β-sheets and N-terminal β-strand conformations. Future investigations will help to confirm and validate these intriguing hypotheses.

While the current study introduces an innovative approach that enables a more comprehensive view of molecular basis for epitope region-specific, chirality-regulated structural features for Aβ fragments, there are still several limitations for further applications. The current work is not a systematic analysis of the amino acid residue-specific contributions to peptide D/L behavior. Future work will combine the iCAP approach described in this study with our previous site-specific method that can pinpoint the amino acid that undergoes isomerization^14^. Further IM-MS studies on the chirality of the full-length Aβ peptide that combines the differential roles of N-termini and C-termini of the peptides would be beneficial for the overall understanding of the chiral effects. To achieve this, several obstacles facing the IM-MS technique have to overcome, including the optimized workflow for obtaining stable and reproducible Aβ signal and the preservation of weak interactions between Aβ-receptors which, however, are beyond the scope of the current study.

In summary, we present an integrative analytical platform, iCAP, that allows us to evaluate the effect of chiral residues in Aβ fragments on their self-assembly and receptor recognition, which could be readily extended to the study of other biological systems. This platform is compatible with any IM-MS setup. The development of such a tool could facilitate future investigation of novel therapeutic treatments for AD as new insights can be obtained via elucidation of the roles of D-isomerized Aβ in early AD development, diagnosis, and prognosis.

## Methods

### Chemicals

Amyloid β peptides with D- and L- forms were purchased from Sangon Biotech Co., Ltd. (Shanghai, China). Other neuropeptide standards were purchased from American Peptide Company (Sunnyvale, CA). [D-Ala]-Deltorphin, [D-Phe]-achatin-I and their all-L forms were synthesized in Biotechnology Center, University of Wisconsin-Madison. The TTR and mTTR protein samples were generously provided by Professor Regina M. Murphy. Other chemicals (including copper acetate salt, ammonium acetate) and protein samples (HSA/BSA) were obtained from Sigma-Aldrich (St. Louis, MO). No further purification was performed for the reagents. All solvents used in this study were of HPLC grade supplied by Sigma-Aldrich (St. Louis, MO). Purified water (conductivity of 18.2 MΩ cm) was obtained from a Milli-Q® Reference System (Millipore Corp., Bedford, MA, USA).

### CCS calibration and calculation

Theoretical CCS values using IMPACT software based on the target PDB file^57^. The TOF-MS was typically operated over the *m/z* range of 400–8000. CCS calibration curves were generated using a previously described protocol, and using literature CCS values derived for use with the Synapt instrument platform ^58,59^.

As with the previous publication^58^, the calibration of travelling-wave IM drift times followed these steps:

1. Prepare calibrant solutions by diluting stocks of melittin, bovine ubiquitin, beta-lactoglobulin and bovine serum albumin in 100 mM ammonium acetate at a concentration of 1–5 μM.
2. Record IM-MS data for ultrafast thermal unfolding proteins at an optimized wave height and velocity to separate the ions.
3. Use precisely the same instrument conditions (including pressures) for all elements downstream of the trapping ion guide to acquire data for the calibrant proteins.
4. Correct calibrant drift times (acquired using a single wave-height value) for mass-dependent flight time, calculated by the Eq. (1) as shown below,
  1 $$t_D^\prime = t_D - \left[ {\frac{{c\sqrt {m/z} }}{{1000}}} \right]$$
  where $t_D^\prime$ is the corrected drift time in ms, *t*_*D*_ is the experimental drift time in ms, *m/z* is the mass-to-charge ratio of the observed ion and *c* is a constant.
5. Take calibrant collision cross-sections (Ω) and correct them for both ion charge state and reduced mass (*μ*) to generate Ω′ by using the Eq. (2),
  2 $${\mathrm{\Omega }}^\prime = {\mathrm{\Omega }}/ \left[\mathrm{charge} \times (1/\mu )^{0.5}\right]$$
6. Create a plot of In *t*′_*D*_ against In Ω′.
7. Fit the plot to a linear relationship of the Eq. (3):
  3 $${\mathrm{ln}}\, \Omega ^\prime = {\mathrm{X}} \times {\mathrm{ln}}\, t_D^\prime + {\mathrm{ln}}\, A$$
  where A is a fit-determined constant and X is referred to as the ‘exponential factor’. The correlation coefficient of the fit achieved in this step should be high (*R*^2^ > 0.98).
8. Re-plot Ω versus a new corrected drift time ($t_D^{\prime\prime}$), where *t*_*D*_ is given by Eq. (4):
  4 $$t_D = \left[t_D^{\prime X} \times \mathrm{charge} \times (1/\mu )^{0.5}\right]$$
9. Use the plot generated in Step 8 to calibrate drift time data for target proteins.

### OpenSPR

We select OpenSPR as a tool for the discrimination of chiral Aβ binding behavior, as OpenSPR™ is a powerful instrument providing in-depth label-free binding kinetics for a variety of different molecular interactions. One of the most common applications of SPR is the analysis and quantification of the interactions between proteins. SPR experiments were performed using a Nicoya Lifesciences OpenSPR system equipped with a COOH chip. Following the start-up procedure found in the OpenSPR™ manual, setup the OpenSPR™ instrument and load a COOH Sensor Chip. The COOH surface was activated following the instructions included in the Amine kit. The ligand was diluted to a concentration of 45 μg/mL into immobilization buffer (provided by Nicoya Lifesciences, pH 4.5) and 100 μL was injected at 20 μL/min for a 5-min interaction time. Aβ receptor, tetrameric transthyretin (TTR), was immobilized using standard procedures. Immobilization was monitored via absorbance change in a standard solution of filtered buffer (PBS, pH 6.8, with 0.005% Tween 20) for 5 min at a flow rate of 50 μl/min. The surface was blocked with an injection of 100 μL of blocking buffer (provided by Nicoya Lifesciences, 1 M ethanolamine, pH 8.5). The flow rate was increased to 50 μL/min, and the analyte was injected at the following concentrations: Aβ C-terminals: 2.4, 4.8, 24, and 48 μM; Aβ (1–40/42): 0.2, 1, 2, 10, and 20 μM. An association time of 150 s and a dislocation time of 400 s were used. The surface was regenerated with an injection of regeneration buffer (HCl 10 mM, pH 2.0) at a speed of 150 μL/min between each analyte injection. The chip was washed in 10 mM HCl (pH 2.0) to remove impurities. Data were collected at a rate of 5 Hz and was single referenced with blank injections. Data were fit to a 1:1 interaction model using the analysis software TraceDrawer.

### Data analysis

To generate the CIU fingerprints, only the data at *m/z* values corresponding to the selected charge state of the precursor ions were selected for analysis. We used the CIUSuite to process CIU data as published previously^30,51^. Once the amount of parent ion was less than five percent of the total signal, the CIU fingerprinting experiments ended. The data were normalized at each voltage through dividing the intensities of ions at each drift time by the maximum ion intensity observed at that voltage.

### Native IM-MS

Each sample of approximately 5 μL was loaded into a home-made nanospray ion source, and a silver wire of 100 μm thickness was inserted into the borosilicate glass needle for high voltage application. For most neuropeptide/DAACP monomer experiments, the concentrations of peptides and Cu^2+^ were set as 10–20 and 150 μM, respectively. For Aβ N-terminal monomer discrimination, this ratio was set as 15 and 20 μM, respectively. For Aβ C-terminal monomer discrimination, this ratio was set as 10 and 50 μM, respectively. All peptide samples were prepared in 10 mM NH_4_OAc (if not otherwise specified). All reactions were monitored after incubation in a water bath at 37 °C for at least three hours. Approximately 5 μL of each sample was loaded into the nanospray source and the MS instrument was run in positive ion mode. Nanospray voltages ranged between 1.0–2.0 kV and the sampling cone was used at 30 V. In typical nanospray experiments, the size of the spray emitter was maintained at ~5 µm. The emitters were pulled from borosilicate glass capillaries using a P-2000 laser-based micropipette puller (Sutter Instruments, Novato, CA, USA). All IM-MS data were collected using Waters Synapt G2 instrument (Waters, Milford, MA, USA). The MS cone temperature was 75 °C. The Synapt instrument was tuned to allow preservation and transmission of native proteins and protein interactions. This typically involved elevated pressures in the source region (~6 mbar), and decreasing all focusing voltages (e.g., cone, extractor, and bias voltages). The traveling-wave ion mobility separator was operated at a pressure of ∼3.5 mbar, and DC voltage waves (30 V wave height traveling at 400 m/s) to generate ion mobility separation. CIU was achieved by increasing the trap CE from 10–170 V with a step voltage of 10 V.

### Reporting summary

Further information on research design is available in the Nature Research Reporting Summary linked to this article.

## Supplementary information

Supplementary Information

Reporting Summary

Source Data

## Data Availability

All data are available from the corresponding author upon reasonable request. The source data underlying Figs. 2c, f, i, l, 3a–d, 4–k, 5a–d, i–l and Table 2 are provided as a Source Data file.
