# Supplementary material for: Molecular basis for chirality-regulated Aβ self-assembly and receptor recognition revealed by ion mobility-mass spectrometry
Source: Nat Commun. 2019 Nov 6;10:5038. doi: 10.1038/s41467-019-12346-8 (PMC6834639; doi:10.1038/s41467-019-12346-8)
Supplement: Supplementary file 1 — Supplementary Information [file 41467_2019_12346_MOESM1_ESM.docx]

**Supplementary Information**

**Molecular Basis for Chirality-Regulated Aβ Self-Assembly and Receptor Recognition Revealed by Ion Mobility-Mass Spectrometry**

Li et al.

**Table of Contents**

**1. Supplementary Figures 1-11**

**2. Supplementary Table 1**

**3. Supplementary Reference**

**1. Supporting Figures**


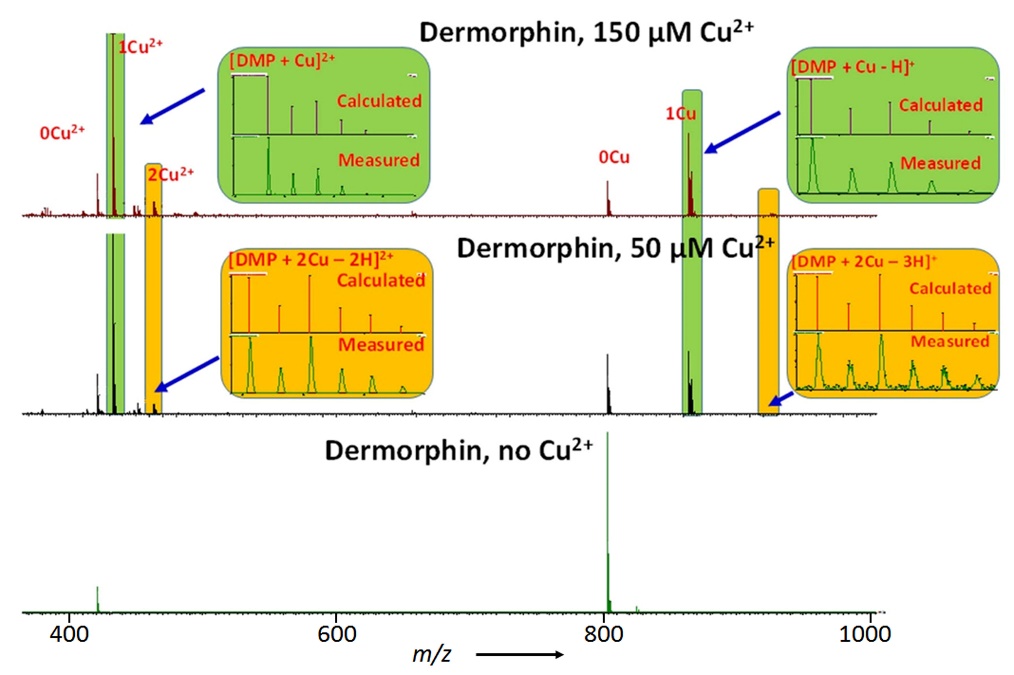


***Supplementary Figure 1.*** Representative MS spectrum for peptide-metal binding. Dermorphin, YAFGYPS, ~20 µM in 10 mM NH_4_OAc.


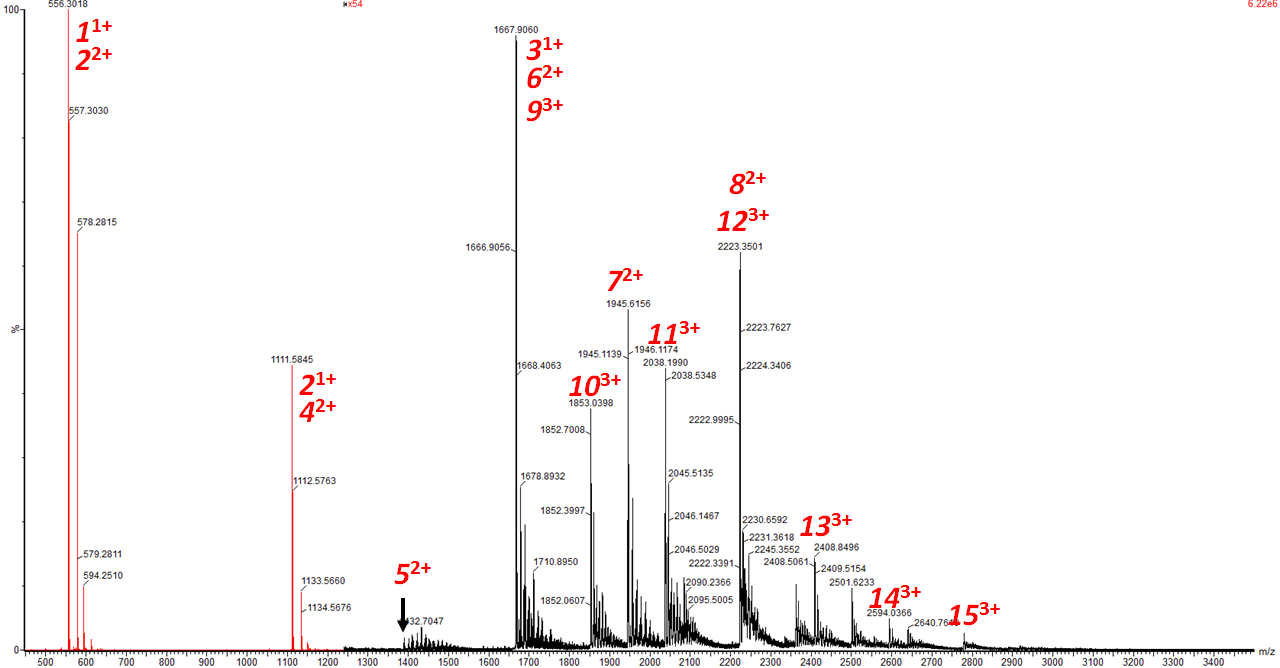


***Supplementary Figure 2.*** Representative MS spectrum for YGGFL oligomerization. YGGFL, 1000 µM. Buffer, 10 mM NH_4_OAc.


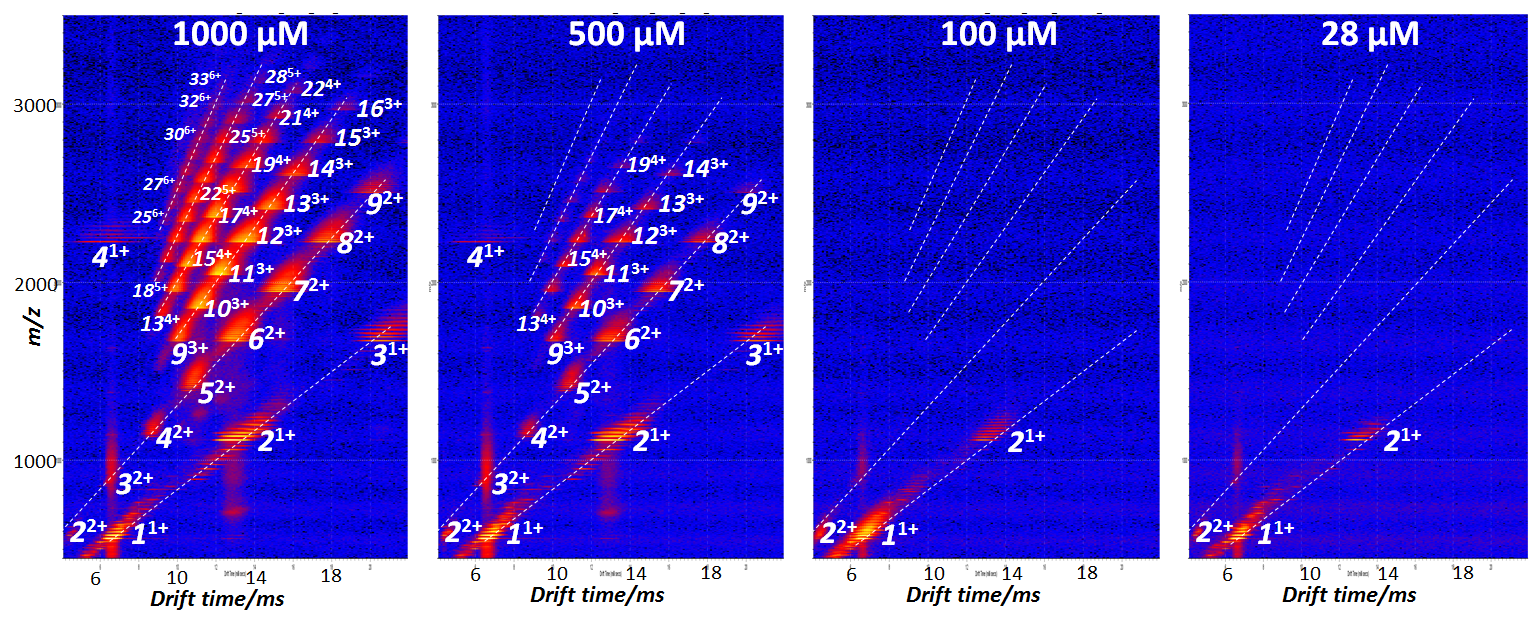


***Supplementary Figure 3.*** Driftscope data for YGGFL oligomerization at various concentrations (1000, 500, 100 and 28 µM). Buffer, 10 mM NH_4_OAc.


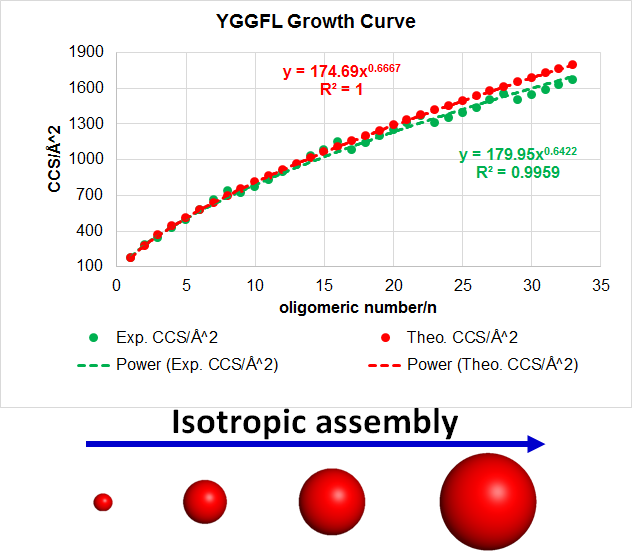


***Supplementary Figure 4.*** Growth curve for YGGFL oligomerization. YGGFL, 1000 µM. Buffer, 10 mM NH_4_OAc.


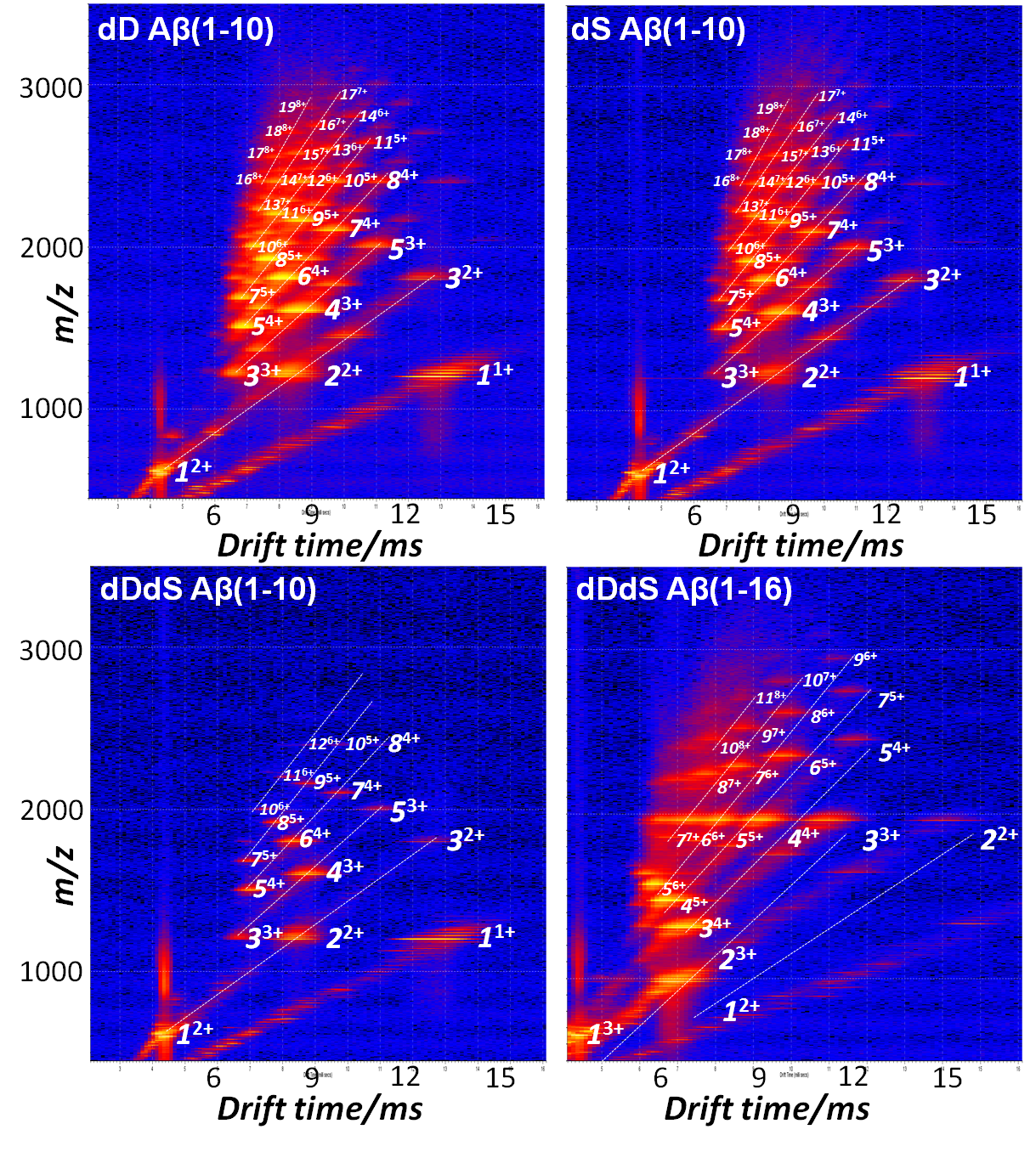


***Supplementary Figure 5.*** Driftscope maps for dD Aβ (1-10), dS Aβ (1-10), dDdS Aβ (1-10) and dDdS Aβ (1-16). Aβ, 600 µM. Buffer, 10 mM NH_4_OAc.


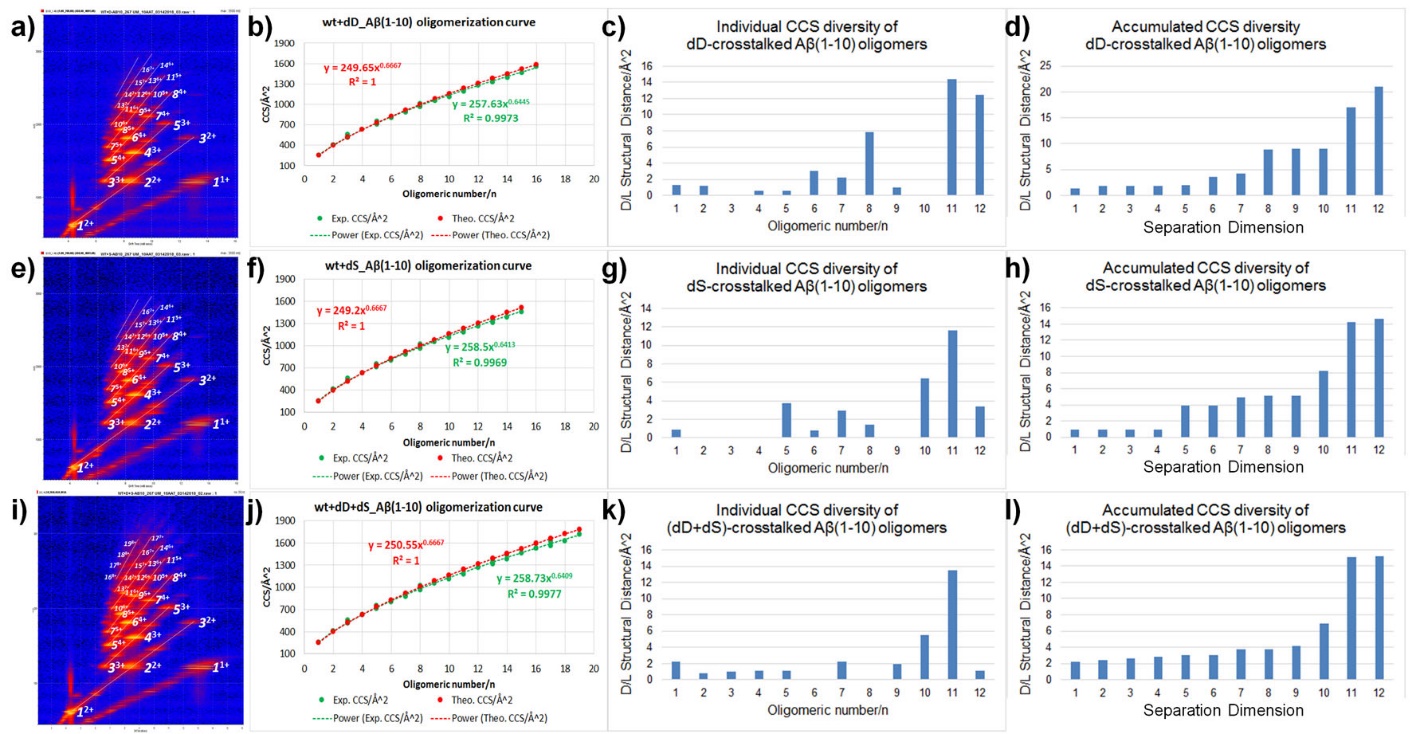


***Supplementary Figure 6.*** The iCAP strategy enables distinguishing crosstalking chiral Aβ oligomerization. Three rows correspond to crosstalking analysis for wild type-dD Aβ (1-10) (**a-d**); wild type-dS Aβ (1-10) (**e-h**) and wild type-dD Aβ (1-10)-dS Aβ (1-10) (**i-l**), respectively. Four columns correspond to representative Driftscope maps, growth curve, individual CCS differences of Aβ oligomers and accumulated DLSDs of Aβ oligomers as calculated by accumulating the CCS differences in third column using functions in **Figure 1**. Buffer, 10 mM NH_4_OAc. Crosstalking experiments, individual Aβ (1-10), 267 µM.


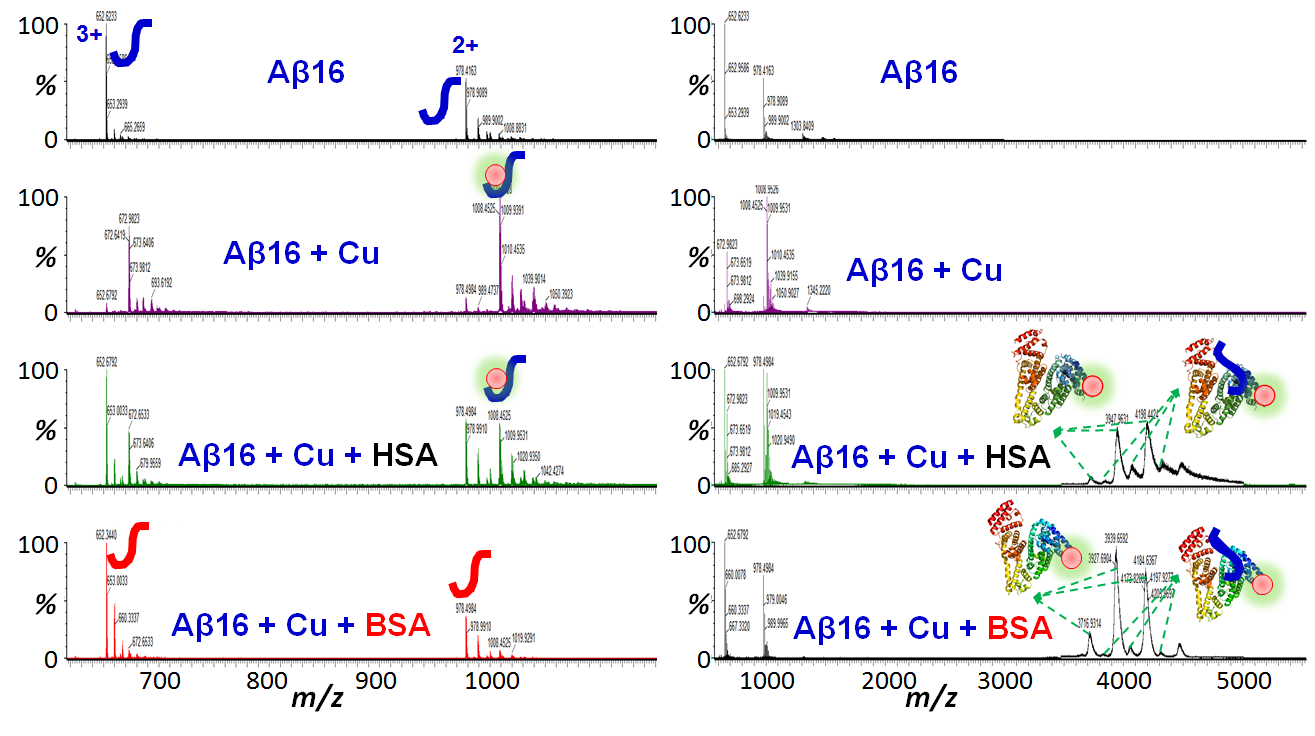


***Supplementary Figure 7.*** The representative mass spectra for triangle interactions of Aβ-Cu-HSA/BSA. All Aβs refer to N-terminal fragment (1-16). Buffer, 100 mM NH_4_OAc. Concentrations: Aβ, 15 µM; Cu^2+^, 20 µM; BSA/HSA, 7.5 µM. Left panel shows the zoomed view of right panel with a certain mass range to visualize the binding ratios change of Cu^2+^ into Aβ upon incubating with receptor HSA/BSA.


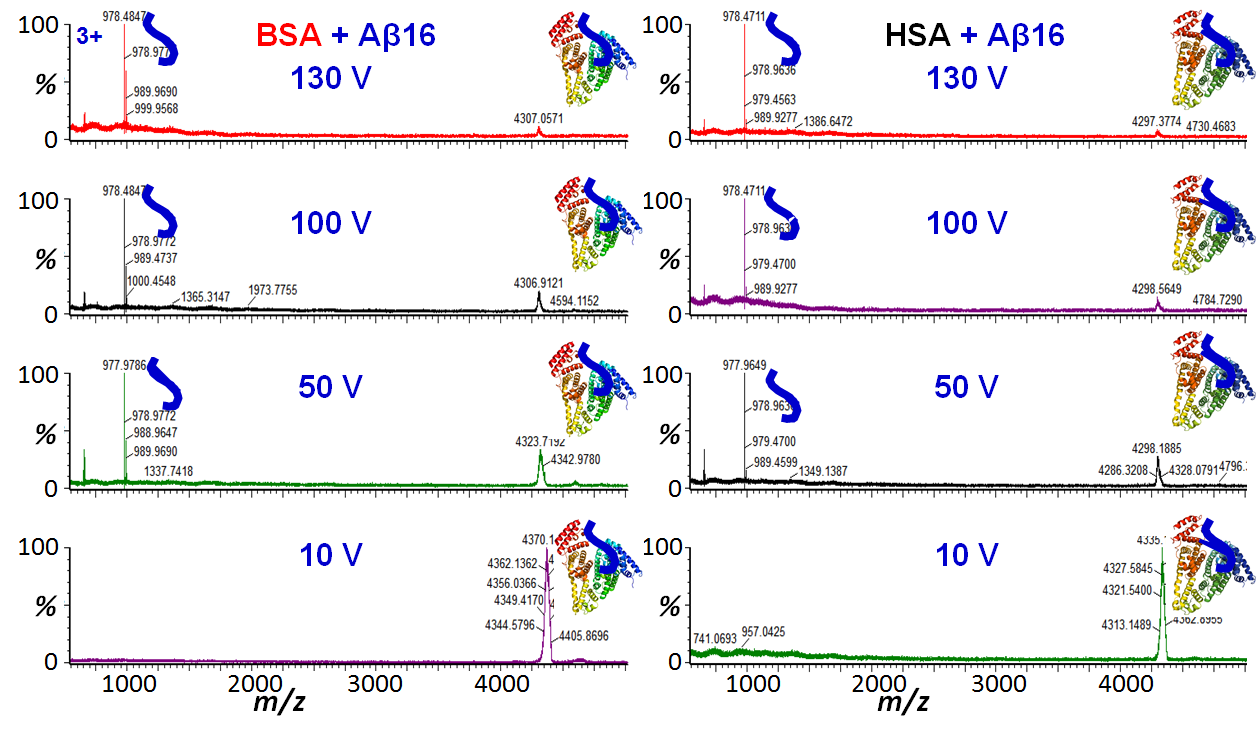


***Supplementary Figure 8.*** Gradual release of Aβ (1-16) from Aβ-HSA/BSA complex upon gas-phase activation.


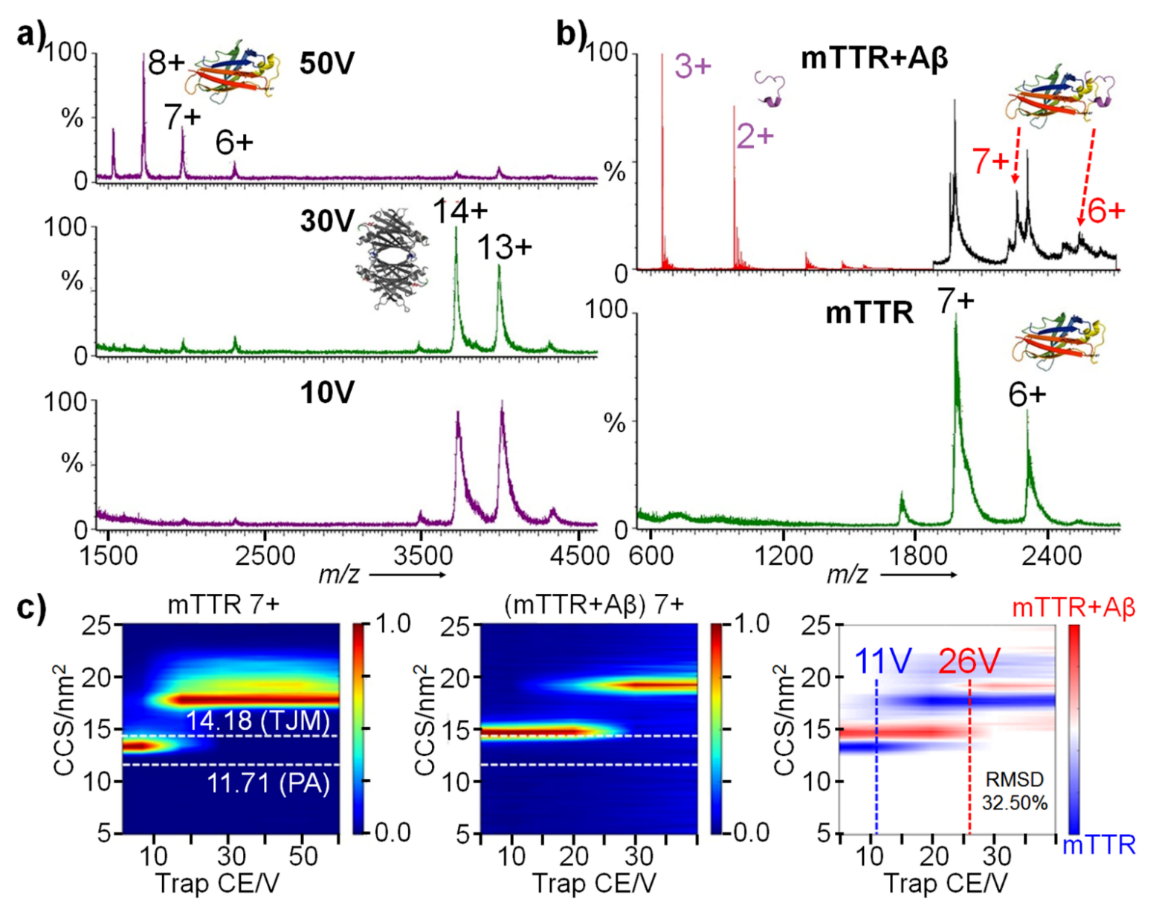


***Supplementary Figure 9.*** CIU/CID-IM-MS provides structural information on TTR-Aβ binding interactions. a) CID analysis of all ions generated from tetrameric TTR. No precursor ion selection was enforced. Trap CE was applied from 10 V, 30 V to 50 V to induce all ion fragmentation. b) Under gentle condition (trap CE 10 V), mTTR binding with Aβ (1-16) was directly observed from IM-MS mass spectrum. c) CIU fingerprints and transitions of mTTR and mTTR-Aβ complex. CIU50 is indicative of conformational transition point. Theoretical CCS values (including PA and TJM) were calculated using IMPACT with PDB entry 1GKO. TTR, ~5 µM. mTTR, ~10 µM. Aβ, 50 µM. Buffer, 50 mM ammonium acetate.


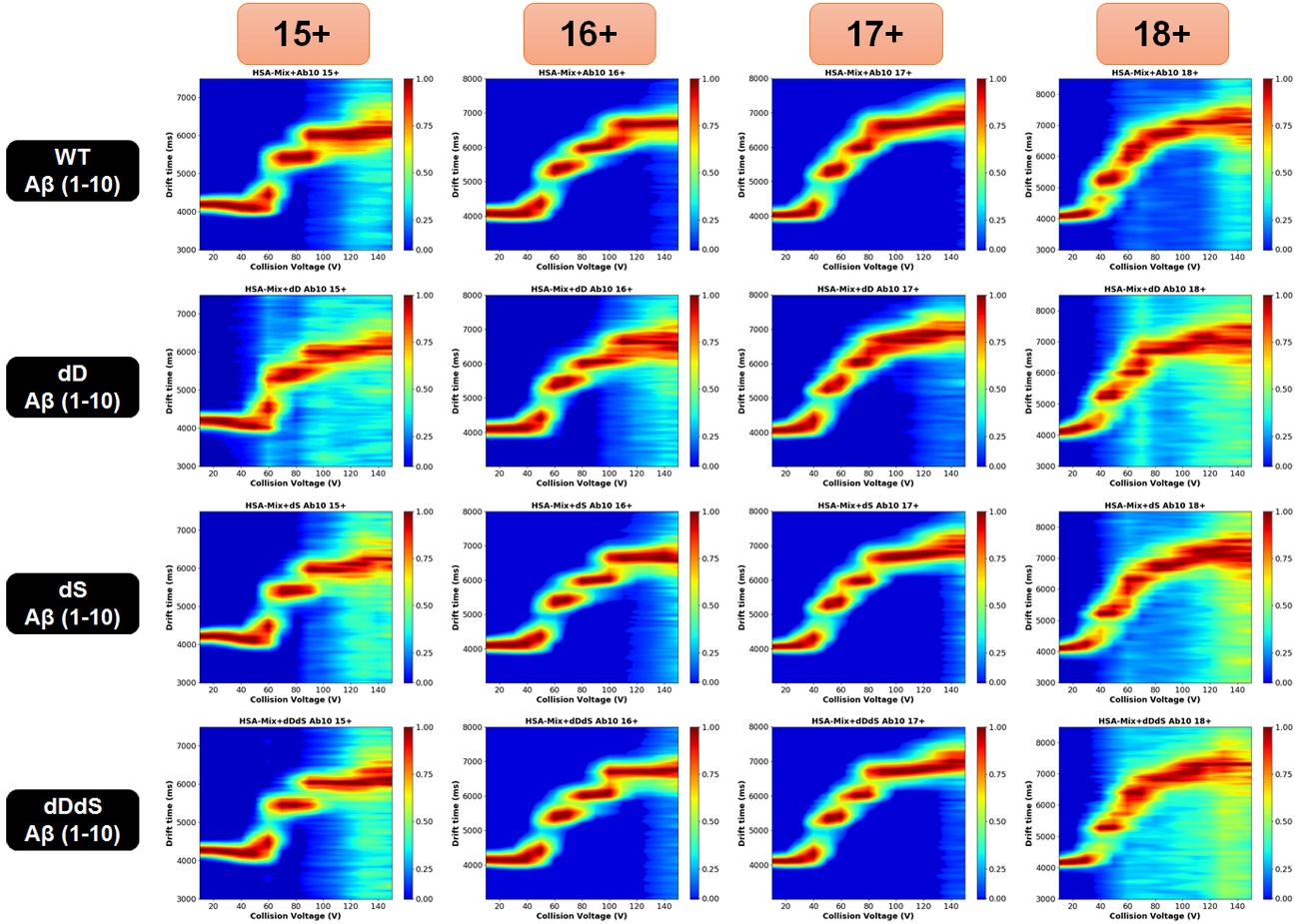


***Supplementary Figure 10.*** Native CIU-IM-MS provides structural information on HSA-Aβ (1-10) fragment binding interactions. Trap CE was applied from 10 V to 150 V to perform CIU. Aβ (1-10) (final conc. 10 µM) was preincubated with Cu^2+^ (final conc. 20 µM) at enough time to ensure complete binding and was monitored using IM-MS. Afterwards, another 2 µL HSA (stock conc. 167 µM) was added to the 38 µL preincubated mixtures (final HSA conc. ~ 8 µM). Buffer, 100 mM ammonium acetate. Only the CIU fingerprints for the HSA-Aβ binding complex are shown here for WT, dD, dS and dDdS Aβ (1-10) with the charge states from 15+ to 18+.


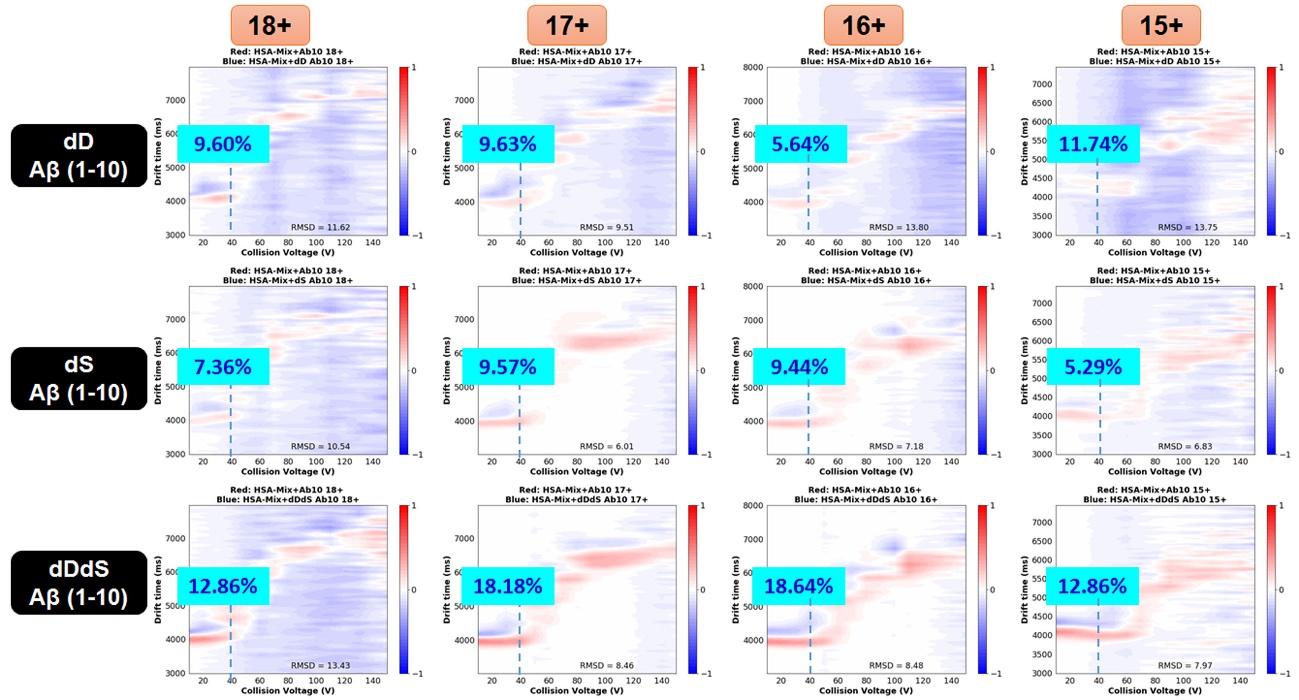


***Supplementary Figure 11.*** CIU difference plots between the complexes of D-isomerized Aβ (1-10)-HSA and the complex of WT Aβ (1-10)-HSA. Trap CE was applied from 10 V to 150 V to perform CIU. Aβ (1-10) (final conc. 10 µM) was preincubated with Cu^2+^ (final conc. 20 µM) for a long enough time to ensure complete binding and was monitored using IM-MS. After that, another 2 µL HSA (stock conc. 167 µM) was added to the 38 µL preincubated mixtures (final HSA conc. ~ 8 µM). Buffer, 100 mM ammonium acetate. Only the CIU fingerprints for HSA-Aβ binding complex are shown here for WT, dD, dS and dDdS Aβ (1-10) with the charge states from 15+ to 18+. The CIU difference plots indicate that the D-isomerization-induced structural changes on the HSA-Aβ (1-10) fragment binding complex follow the order: dDdS > dD > dS. (Note that 16+ charge state has exception with dS > dD for RMSD below 40 V although this value for the whole unfolding energy range is still following the trend with dD > dS: 18+, 11.62% (dD) vs 10.54% (dS); 17+, 9.51% (dD) vs 6.01% (dS); 16+, 13.80% (dD) vs 7.18% (dS) and 15+, 13.75% (dD) vs 6.83% (dS).).

**Supplementary Table 1: Protein Molecular Weight (MW) Information in This Study**

| Protein ID | Expected MW | Experimental MW^+^ |
| --- | --- | --- |
| HSA | 66,437 Da | 66,875 ± 33 Da |
| BSA | 66,463 Da | 66,732 ± 15 Da |
| mTTR* | 13,800 Da | 13,846 ± 9 Da |
| TTR | 55,200 Da | 55,820 ± 47 Da |

*, mTTR, F87M/L110M mutation of monomeric TTR. For more information, please refer to: Pate et al. Transthyretin Mimetics as Anti-beta-Amyloid Agents: A Comparison of Peptide and Protein Approaches. ^1^

^+^, ± represents SD.

**Supplementary References**

1. Pate, K.M., Kim, B.J., Shusta, E.V. & Murphy, R.M. Transthyretin Mimetics as Anti-beta-Amyloid Agents: A Comparison of Peptide and Protein Approaches. *ChemMedChem* **13**, 968-979 (2018).
